# The relationship between vitamin A and myopia: A population-based study

**DOI:** 10.1371/journal.pone.0316438

**Published:** 2025-01-24

**Authors:** Yeo Jin Lee, Donghyun Jee

**Affiliations:** 1 Department of Ophthalmology, Seoul St. Mary’s Hospital, College of Medicine, The Catholic University of Korea, Seoul, Republic of Korea; 2 Department of Ophthalmology and Visual Science, Eunpyeong St. Mary’s Hospital, College of Medicine, The Catholic University of Korea, Seoul, Republic of Korea; 3 Department of Ophthalmology and Visual Science, St. Vincent’s Hospital, College of Medicine, Catholic University of Korea, Suwon, Republic of Korea; Instituto Superior de educação e Ciencias, ISEC Lisboas, PORTUGAL

## Abstract

**Purpose:**

We sought to evaluate the relationship between blood vitamin A levels and myopia in adults aged ≥20 years in Korea.

**Methods:**

We collected data of 15,899 participants aged ≥20 years from the Korean National Health and Nutrition Examination Survey. Participants underwent refraction tests to identify myopia and high myopia, and their blood pressure and obesity levels were measured. Blood tests were conducted to assess vitamin A, fasting blood glucose, triglyceride, and total cholesterol levels. Blood vitamin A levels were classified into quartiles.

**Results:**

After adjusting for confounding variables like age, sex, income, education, hypertension, diabetes, and obesity, the odds ratio (OR) of blood vitamin A in the second quartile for myopia was 0.66, while the OR in the fourth quartile was 0.74 (*P* for trend < 0.001). Among women, the ORs for myopia in the second and third quartiles of blood vitamin A levels were 0.48 (95% confidence interval [CI], 0.35–0.66) and 0.67 (95% CI, 0.49–0.90), respectively (*P* for trend < 0.001). In men, the ORs for high myopia in the second, third, and fourth quartiles of blood vitamin A levels were 0.05 (95% CI, 0.004–0.58), 0.15 (95% CI, 0.024–0.91), and 0.05 (95% CI, 0.008–0.364), respectively (*P* for trend < 0.001).

**Conclusion:**

An inverse relationship was observed between higher blood vitamin A levels and the prevalence of myopia. Notably, higher blood vitamin A levels were associated with a lower prevalence of high myopia in men and a lower prevalence of myopia in women.

## Introduction

Recent increases in reading, computer, and smartphone use have led to a significant rise in the incidence of myopia. In 2000, the global myopic population was estimated to total 1.4 billion, and it is predicted to reach 4.8 billion by 2050 [1]. High myopia can lead to complications like retinal detachment, glaucoma, cataracts, and myopic macular degeneration, ultimately resulting in vision impairment [2]. The causes of myopia have been studied extensively. The development of myopia is influenced by a combination of genetic and environmental factors. Genes like *PAX6* and *GJD2* are strongly associated with myopia, and studies have shown that children of myopic parents are more likely to develop myopia themselves [3]. Regarding environmental factors, several studies have investigated the correlation between outdoor activity time and myopia incidence. It has been confirmed that exposure to sunlight stimulates the secretion of dopamine, which inhibits eye growth and helps prevent myopia [4–6]. Additionally, prolonged near-work activities, such as reading, computer use, and smartphone use, are major risk factors for myopia [7]. Higher levels of education, which are associated with these activities, have also been significantly linked to myopia [8, 9]. Children raised in urban areas have been found to have a greater incidence of myopia compared to those raised in rural areas, likely due to differences in outdoor activity time [8, 10].

Chemical signals such as all-trans retinoic acid (atRA) also play a crucial role in regulating eye growth [11]. AtRA is a metabolite of vitamin A, which is considered essential for eye health. One of the most important functions of vitamin A is its involvement in the visual cycle through the conversion between all-trans-retinal and 11-cis-retinal. Vitamin A deficiency can result in night blindness due to impaired rhodopsin regeneration. Additionally, vitamin A acts as an antioxidant, protecting eye tissues from oxidative stress [12]. Vitamin A is also vital for the maintenance and differentiation of epithelial cells, including those in the cornea and conjunctiva [13], and it supports the immune system, reducing the risk of eye infections that can impair vision [14].

Although vitamin A plays important roles in eye health, its relationship with myopia is not well-supported by current research. One study analyzed the degree of myopia in adolescents and young adults based on vitamin A intake but found no significant relationship [15]. Another study, which investigated the risk factors for myopia in individuals aged 12–25 years, found that high levels of serum vitamin A were associated with an increased prevalence of high myopia [16]. However, the number of relevant studies is extremely limited, and their results are inconsistent and restricted, pertaining largely to adolescents and young adults.

Therefore, we conducted a large-scale epidemiological study to examine the relationship between serum vitamin A level and myopia in South Korea. This study used data from the Korea National Health and Nutrition Examination Survey (KNHANES), which assesses adults aged ≥20 years in South Korea, to evaluate the association between vitamin A and myopia.

## Methods

**Fig 1:**
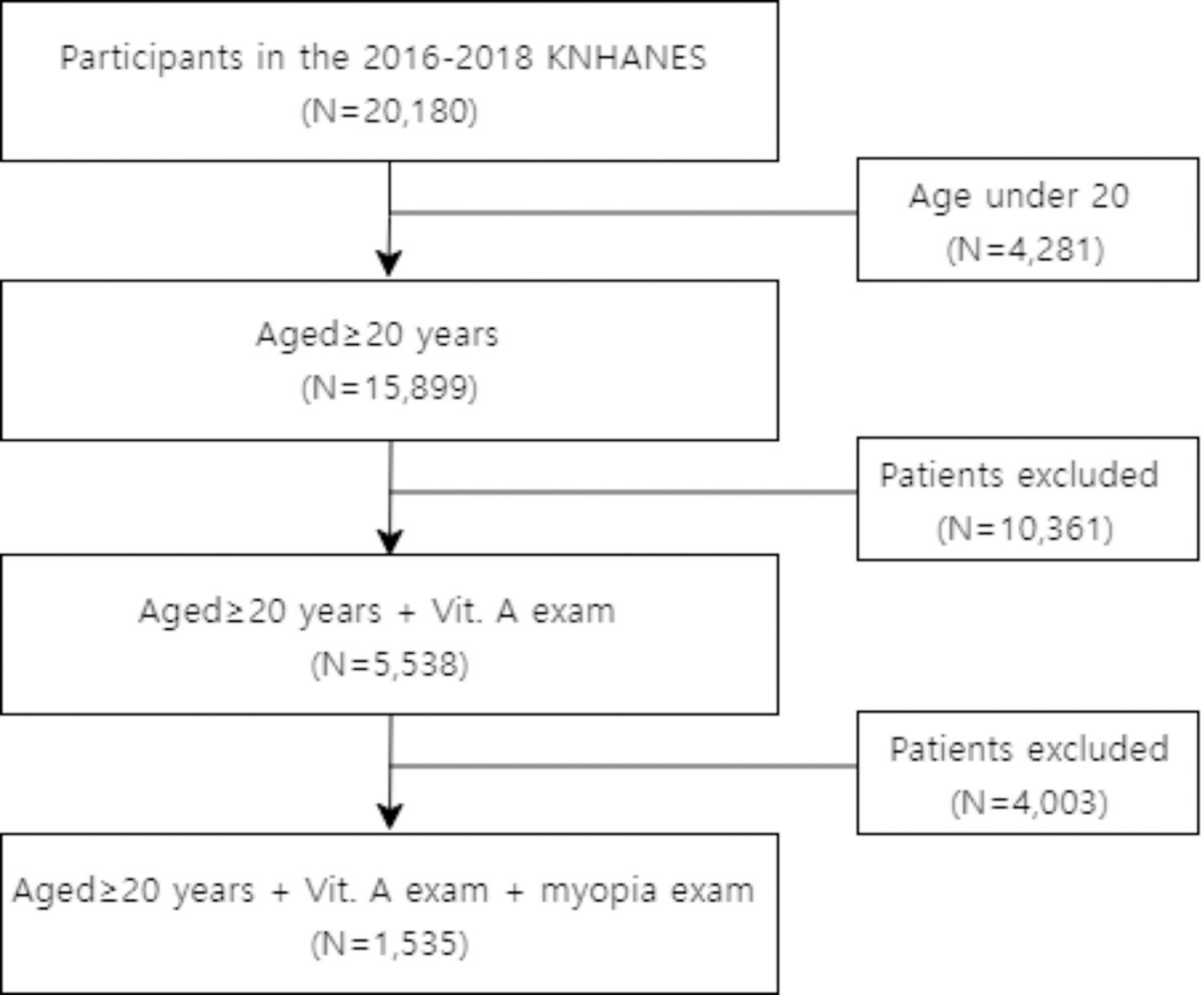
Flow chart presenting the selection of study participants.

This study used data extracted from the 2016–2018 KNHANES. We accessed the data for research purposes from January 1, 2024, to March 31, 2024. During or after data collection, the authors did not have access to information that could identify individual participants. KNHANES is a population-based survey in Korea, using statistical, multi-level, and clustered sampling methods. Informed consent for the use of clinical records was obtained from all participants. A total of 20,180 individuals were selected, with 15,899 of them being >20 years old. Among the selected participants, 10,361 individuals who did not undergo a vitamin A test and 4,003 individuals who did not undergo a myopia test were excluded, resulting in a final sample of 1,535 individuals (Fig 1). This study was approved by the institutional review board (IRB) of Seoul St. Mary’s Hospital, The Catholic University of Korea, and all methods were performed in accordance with the principles of the Declaration of Helsinki (IRB no. VC22ZESI0175).

Participants were interviewed to collect the details of sex, age, body mass index (BMI), educational level, and income. BMI values were classified into six categories based on thresholds of 18.5, 23.0, 25.0, 30.0, and 35.0 kg/m^2^. Educational level was classified into four categories, as follows: elementary school or less, middle school graduate, high school graduate, and university graduate or higher. Also, income was classified into four categories based on monthly income, as follows: <1 million Korean won (KRW), 1–2 million KRW, 2–3 million KRW, and >3 million KRW.

Blood pressure was measured three times in 5-min intervals using a mercury sphygmomanometer (Baumanometer; W.A. Baum Co., Copiague, NY, USA), and the average of these measurements was used for analysis. Prehypertension was defined as a systolic blood pressure of 120–139 mmHg or a diastolic blood pressure of 80–89 mmHg. Hypertension was defined as a systolic blood pressure of ≥140 mmHg, a diastolic blood pressure of ≥90 mmHg, or the use of prescribed antihypertensive medication [17].

Blood tests were conducted after 10–12 h of fasting, and fasting blood glucose, triglycerides, and total cholesterol were measured using a Hitachi automatic analyzer 7600 (Hitachi, Ltd., Tokyo, Japan). Diabetes was defined as a fasting blood glucose level of ≥126 mg/dL, the use of diabetes medication, or insulin injection. The presence of a “fasting blood sugar disorder” was defined by a fasting blood glucose level of 100–125 mg/dL, while “normal” blood sugar was defined by a fasting blood glucose level of ≤100 mg/dL. Serum vitamin A levels were measured using the Agilent 1200 high-performance liquid chromatograph (Agilent Technologies, Santa Clara, CA, USA) and divided into the following quartiles: level 1, serum vitamin A < 0.39 mg/L; level 2, 0.39 mg/L ≤ serum vitamin A < 0.49 mg/L; level 3, 0.49 mg/L ≤ serum vitamin A < 0.62 mg/L; and level 4, serum vitamin A ≥ 0.62 mg/L.

Refraction examinations were performed using the RC-800 auto refractometer (TOMEY Co., Nagoya, Japan) without cycloplegia. Myopia was defined by a spherical equivalent (sum of the spherical error and half of the cylindrical error) of ≤−0.5 D, and high myopia was defined by a spherical equivalent of ≤−5.0 D. ^1^ Statistical analysis was performed using SPSS (ver. 18.0; IBM Corporation, Armonk, NY, USA), with *P* < 0.05 considered statistically significant. Logistic regression analysis was performed to evaluate the relationship between serum vitamin A and myopia. Unadjusted values were defined as “crude,” values adjusted for age and sex were defined as “Model 1,” and values adjusted for age, sex, income, education level, hypertension, diabetes, and BMI were defined as “Model 2.”

In this study, odds ratios (OR) were categorized and interpreted as follows: OR between 0.9 and 1.1 was defined as trivial, OR between 1.1–1.5 or 0.7–0.9 as mild, OR between 1.5–3.0 or 0.4–0.7 as moderate, and OR greater than 3.0 or less than 0.4 as high. If the confidence interval of the OR overlapped with 1, it was interpreted as not statistically significant.

## Results

The demographic characteristics of the participants are summarized in Table 1. The proportion of men among those with myopia was significantly greater at 49.9% compared to 47.1% among those without myopia (*P* = 0.001). Subjects with myopia were significantly younger, with a mean age of 53.3 years compared to that of 61.7 years for those without myopia (*P* < 0.001). Those with myopia also had lower systolic blood pressure and higher diastolic blood pressure values (*P* < 0.001 and *P* < 0.001, respectively), and fasting blood glucose levels were also lower in the myopia group (*P* < 0.001). A significant association between myopia and the level of education was also noted (*P* < 0.001), with the university graduation rate being notably high (45.5%) among individuals with myopia. The mean blood vitamin A level showed a marginal association with myopia (*P* = 0.061). Meanwhile, myopia had a significant association with blood vitamin A levels when they were divided into quartiles (*P* = 0.017). Hypertension, diabetes, and BMI were all significantly associated with myopia (*P* < 0.01, *P* < 0.01, and *P* = 0.030, respectively). Finally, total cholesterol and triglycerides were also significantly associated with myopia (*P* < 0.01 and *P* = 0.004, respectively).

**Table 1:** Clinical and demographic characteristics, according to myopia, as reported in the Korean National Health and Nutrition Examination Survey 2016–2018. Value is described as weighted means or weighted frequency (%) with standard errors. BMI: Body mass index.

Characteristics	without myopia	Myopia	*p*	Participants
**Male (%)**	47.1 (0.6)	49.9 (0.7)	.001	48.4 (0.5)
**Age (yrs)**	61.7 (0.2)	53.3 (0.2)	< .001	57.5 (0.2)
**Systolic blood pressure (mmHg)**	122.3 (0.3)	118.9 (0.3)	< .001	120.6 (0.2)
**Diastolic blood pressure (mmHg)**	75.3 (0.1)	77.3 (0.2)	< .001	76.3 (0.1)
**Fasting glucose (mg/dL)**	104.8 (0.3)	102.6 (0.4)	< .001	103.7 (0.3)
**Vitamin A (mg/L)**	0.60 (0.0)	0.59 (0.0)	.061	0.59 (0.0)
**Education (%)**			< .001	
** Elementary school**	30.4 (0.8)	12.8 (0.5)		22.1 (0.6)
** Middle school**	15.9 (0.5)	8.1 (0.4)		12.2 (0.4)
** High school**	31.8 (0.6)	33.6 (0.8)		32.6 (0.5)
** University**	21.9 (0.8)	45.5 (1.2)		33.1 (0.9)
**Monthly income (%)**			.551	
** <1 million KRW**	23.6 (0.6)	23.6 (0.8)		23.6 (0.6)
** 1–2 million KRW**	26.0 (0.7)	24.8 (0.7)		25.4 (0.5)
** 2–3 million KRW**	24.8 (0.6)	25.7 (0.9)		25.2 (0.6)
** >3 million KRW**	25.6 (0.8)	25.9 (0.9)		25.7 (0.7)
**Vitamin A (%)**			.017	
** Vit A < 0.39**	12.3 (0.9)	16.0 (1.0)		14.2 (0.7)
** 0.39 ≤ Vit A < 0.49**	25.8 (1.2)	22.7 (1.1)		24.2 (0.9)
** 0.49 ≤ Vit A < 0.62**	27.0 (1.0)	27.1 (1.3)		27.1 (0.8)
** Vita A ≥ 0.62**	34.9 (1.3)	34.2 (1.2)		34.6 (0.9)
**Hypertension (%)**			< .001	
** Normal**	31.2 (0.7)	41.5 (0.9)		36.0 (0.6)
** Prehypertension**	25.0 (0.6)	26.2 (0.6)		25.6 (0.5)
** Hypertension**	43.8 (0.6)	32.3 (0.8)		38.4 (0.5)
**Diabetes severity (%)**			< .001	
** Normal**	51.8 (0.6)	59.1 (0.8)		55.2 (0.6)
** Fasting blood sugar disorder**	30.4 (0.7)	29.2 (0.7)		29.8 (0.5)
** Diabetes**	17.9 (0.5)	11.7 (0.5)		15.0 (0.4)
**Body mass index (kg/m** ^**2**^ **)**			.030	
** BMI < 18.5**	2.2 (0.2)	2.7 (0.2)		2.4 (0.1)
** 18.5 ≤ BMI < 23.0**	35.6 (0.7)	37.5 (0.8)		36.5 (0.5)
** 23.0 ≤ BMI < 25.0**	25.7 (0.6)	24.6 (0.7)		25.2 (0.5)
** 25.0 ≤ BMI < 30.0**	32.1 (0.6)	31.4 (0.7)		31.7 (0.5)
** 30.0 ≤ BMI < 35.0**	4.3 (0.3)	3.5 (0.3)		3.9 (0.2)
** BMI ≥ 35**	0.2 (0)	0.4 (0.1)		0.3 (0.1)
**Total cholesterol (mg/dL)**	191.9 (0.6)	196.7 (0.6)	< .001	194.3(0.5)
**Triglyceride (mg/dL)**	142.1 (1.9)	151.3 (2.2)	.004	146.7 (1.4)

Table 2 describes the distribution of participants according to the quartiles of blood vitamin A levels. Table 3 presents the odds ratios for myopia according to the quartiles of vitamin A levels across different models. Logistic regression analysis was conducted using a step-by-step approach, adding variables one by one from Model 1 to Model 2. The R^2^ value increased from 0.005 in the Crude model to 0.179 when sex and age (Model 1) were included, and further increased to 0.187 in Model 2, which included all variables. In model 2, which was adjusted for age, sex, income, education, hypertension, diabetes, and obesity, participants in the second quartile of blood vitamin A levels had a moderate odds ratio (OR) of 0.66 compared to those in the first quartile (95% confidence interval [CI], 0.52–0.83). Separately, those in the fourth quartile had a mild OR of 0.74 compared to those in the first quartile (95% CI, 0.56–0.96) (*P* for trend < 0.001). Therefore, a significant relationship was observed indicating that higher blood vitamin A levels are associated with a lower prevalence of myopia.

**Table 2:** Distribution of myopia according to quartile levels of blood vitamin A in representative Korean adults aged 20 years or older. Value is described as estimated value (%) or standard error (%) or unweighted frequency.

		myopia status		
Quartile blood vitamin A level (mg/L)		Without myopia	myopia	Total
Quartile level 1 **(**<0.39)	Estimated value	42.7%	57.3%	100.0%
	Standard error	2.5%	2.5%	0.0%
Quartile level 2 **(**0.39–0.49)	Estimated value	52.5%	47.5%	100.0%
	Standard error	1.9%	1.9%	0.0%
Quartile level 3 (0.49–0.62)	Estimated value	49.2%	50.8%	100.0%
	Standard error	1.6%	1.6%	0.0%
Quartile level 4 (≥0.62)	Estimated value	49.8%	50.2%	100.0%
	Standard error	1.5%	1.5%	0.0%
Total	Estimated value	49.3%	50.7%	100.0%
	Standard error	0.9%	0.9%	0.0%

**Table 3:** Adjusted odds ratio of myopia stratified according to quartile levels of blood vitamin A in representative Korean adults aged 20 years or older. Logistic regression analysis was performed by adding variables step by step to investigate the individual effect of each variable on the outcome. Value is described as odd ratio (95% confidence intervals). Model 1: adjusted for age and sex. Model 2: adjusted for age, sex, income, education, hypertension, diabetes, and obese.

Quartile blood vitamin A level (mg/L)	Crude	Model 1	+ income	+education	+hypertension	+diabetes	Model 2 (+obese)
Quartile level 1 **(**<0.39)	1.0 (reference)	1.0 (reference)	1.0 (reference)	1.0 (reference)	1.0 (reference)	1.0 (reference)	1.0 (reference)
Quartile level 2 **(**0.39–0.49)	0.67 (0.53–0.86)	0.66 (0.53–0.83)	0.67 (0.53–0.83)	0.66 (0.52–0.84)	0.67 (0.53–0.85)	0.67(0.53–0.84)	0.66 (0.52–0.83)
Quartile level 3 (0.49–0.62)	0.77 (0.60–0.99)	0.81 (0.63–1.03)	0.81 (0.63–1.03)	0.84 (0.65–1.09)	0.84 (0.65–1.08)	0.83 (0.65–1.06)	0.80 (0.63–1.03)
Quartile level 4 (≥0.62)	0.75 (0.59–0.96)	0.75 (0.58–0.96)	0.75 (0.58–0.96)	0.73 (0.56–0.96)	0.72 (0.55–0.94)	0.75 (0.58–0.98)	0.74 (0.56–0.96)
P for trend	0.019	< .001	< .001	< .001	< .001	< .001	< .001
R^2^ (Nagelkerke)	0.005	0.179	0.185	0.193	0.194	0.188	0.187

Table 4 compares the ORs for myopia according to the quartiles of blood vitamin A levels for men and women separately. Among men, there were no statistically significant values across all quartiles. For women, the ORs for the second and third quartiles were 0.48 (moderate, 95% CI, 0.35–0.66) and 0.67 (moderate, 95% CI, 0.49–0.90), respectively, compared to the first quartile (*P* for trend < 0.001).

**Table 4:** Odds ratio of myopia in male and female stratified according to the quartile category of vitamin A in the blood in representative Korean adults aged 20 years or older, after adjusting for age, income, education, hypertension, diabetes and obese. Value is described as odds ratio (95% confidence intervals).

Quartile blood vitamin A level (mg/L)	Odds ratio
**Male**	
Quartile level 1 **(**<0.39)	1.0 (reference)
Quartile level 2 **(**0.39–0.49)	1.28 (0.70–2.33)
Quartile level 3 (0.49–0.62)	1.16 (0.66–2.03)
Quartile level 4 (≥0.62)	1.00 (0.58–1.73)
P for trend	< .001
**Female**	
Quartile level 1 **(**<0.39)	1.0 (reference)
Quartile level 2 **(**0.39–0.49)	0.48 (0.35–0.66)
Quartile level 3 (0.49–0.62)	0.67 (0.49–0.90)
Quartile level 4 (≥0.62)	0.72 (0.50–1.04)
P for trend	< .001

Subsequently, analysis revealed a significantly smaller sample size of 50 individuals with high myopia compared to 714 individuals with myopia. The distribution of participants with high myopia according to the quartiles of blood vitamin A levels is shown in Table 5. The ORs for high myopia did not show significant differences in the crude, Model 1, or Model 2 subsets (Table 6).

**Table 5:** Distribution of high myopia according to quartile levels of blood vitamin A in representative Korean adults aged 20 years or older. Value is described as estimated value (%) or standard error (%) or unweighted frequency.

		myopia status		
Quartile blood vitamin A level (mg/L)		Without high myopia	High myopia	Total
Quartile level 1 **(**<0.39)	Estimated value	96.8%	3.2%	100.0%
	Standard error	0.8%	0.8%	0.0%
Quartile level 2 **(**0.39–0.49)	Estimated value	97.4%	2.6%	100.0%
	Standard error	0.6%	0.6%	0.0%
Quartile level 3 (0.49–0.62)	Estimated value	95.8%	4.2%	100.0%
	Standard error	0.6%	0.6%	0.0%
Quartile level 4 (≥0.62)	Estimated value	97.0%	3.0%	100.0%
	Standard error	0.5%	0.5%	0.0%
Total	Estimated value	96.7%	3.3%	100.0%
	Standard error	0.3%	0.3%	0.0%

**Table 6:** Adjusted odds ratio of high myopia stratified according to quartile levels of blood vitamin A in representative Korean adults aged 20 years or older. Value is described as odd ratio (95% confidence intervals). Model 1: adjusted for age and sex. Model 2: adjusted for age, sex, income, education, hypertension, diabetes and obese.

Quartile blood vitamin A level (mg/L)	Crude	Model 1	Model 2
Quartile level 1 **(**<0.39)	1.0 (reference)	1.0 (reference)	1.0 (reference)
Quartile level 2 **(**0.39–0.49)	0.81 (0.38–1.71)	0.96 (0.43–2.14)	0.79 (0.33–1.93)
Quartile level 3 (0.49–0.62)	1.33(0.73–2.43))	1.72 (0.83–3.55)	1.87 (0.86–4.09)
Quartile level 4 (≥0.62)	0.93 (0.48–1.78)	1.25 (0.51–3.03)	0.89 (0.30–2.58)
P for trend	.238	< .001	< .001

However, when analyzing the ORs for high myopia by sex, there was no significant difference for women, but a marked difference was observed for men. Men in the second quartile of blood vitamin A levels had an OR of 0.05 compared to those in the first quartile (high, 95% CI, 0.004–0.58), those in the third quartile had an OR of 0.15 compared to those in the first quartile (high, 95% CI, 0.024–0.91), and those in the fourth quartile had an OR of 0.05 compared to those in the first quartile (high, 95% CI, 0.008–0.364) (*P* for trend < 0.001) (Table 7).

**Table 7:** Odds ratio of high myopia in male and female stratified according to the quartile category of vitamin A in the blood in representative Korean adults aged 20 years or older, after adjusting for age, income, education, hypertension, diabetes and obese. Value is described as odds ratio (95% confidence intervals).

Quartile blood vitamin A level (mg/L)	Odds ratio
**Male**	
Quartile level 1 **(**<0.39)	1.0 (reference)
Quartile level 2 **(**0.39–0.49)	0.05 (0.004–0.58)
Quartile level 3 (0.49–0.62)	0.15 (0.024–0.91)
Quartile level 4 (≥0.62)	0.05 (0.008–0.364)
P for trend	< .001
**Female**	
Quartile level 1 **(**<0.39)	1.0 (reference)
Quartile level 2 **(**0.39–0.49)	1.17 (0.51–2.69)
Quartile level 3 (0.49–0.62)	2.73 (1.58–4.72)
Quartile level 4 (≥0.62)	1.37 (0.43–4.36)
P for trend	< .001

## Discussion

The study confirmed that blood vitamin A levels affect myopia. Higher blood vitamin A levels were associated with a lower prevalence of myopia, suggesting that vitamin A may act as a preventive factor against myopia. Notably, higher vitamin A levels were linked to lower prevalence rates of myopia in women and high myopia in men.

The reason for the reduced myopia prevalence in connection with higher blood vitamin A levels can be explained based on previous studies. There are various research findings pertaining to ocular growth regulation and retinoic acid. AtRA, a metabolite of retinol (vitamin A), is synthesized in the choroid in response to visual stimuli and transported to the sclera by the apoA-1 protein [11, 18, 19]. It is hypothesized that atRA regulates the transcription of various genes in the sclera, leading to remodeling of the extracellular matrix, changes in eye size, and refractive errors [20]. Several animal studies have revealed that eye growth is partially regulated by a process of "emmetropization" to minimize refractive errors. When wearing convex lenses (plus lenses) or recovering from form-deprivation myopia, the axial length of the eye decreases until it matches the focal plane [21, 22]. Additionally, studies in chicks have confirmed that synthesis of atRA in the choroid increases while wearing convex lenses or recovering from form-deprivation myopia [11]. The observed rate of increase in choroidal atRA synthesis is similar to the rate of decrease in scleral proteoglycan synthesis [23]. Therefore, it can be assumed that increased atRA in the choroid reduces proteoglycan synthesis in the sclera, leading to slower scleral growth.

In this study, higher vitamin A levels showed a moderate OR, indicating a moderate association with the prevalence of myopia in women, but no association was observed in men. Conversely, for the prevalence of high myopia, higher vitamin A levels were not associated with women but showed a high OR and a strong association in men. These findings suggest that vitamin A may have differential effects depending on gender. Sufficient vitamin A levels could help prevent mild to moderate myopia in women, reducing the need for glasses. However, it can be inferred that in men, it may help prevent high myopia, which carries a risk of blindness. Nevertheless, this remains a hypothesis, and further research is needed.

Several reasons may explain why such differences exist between genders. High myopia is associated with more significant thinning and atrophy of the choroid compared to mild myopia, resulting in reduced choroidal blood flow [24]. It is known that men tend to have thicker choroids and larger blood vessels compared to women [25]. This difference suggests that the concentration of atRA synthesized in the choroid may be more responsive in men, although further research is needed to confirm this hypothesis.

Differences due to sex hormones can also be considered. Animal studies have shown that an increase in estrogen can boost the synthesis of retinoic acid [26]. However, in this study, the average blood vitamin A level in men (0.56 mg/L) was greater than that in women (0.45 mg/L). There are studies indicating that retinoic acid inhibits testosterone in lacrimal gland cells [27], but the reverse—how testosterone affects retinoic acid—has not yet been researched. Additionally, the use of contraceptives or hormone replacement therapy in women might also influence these dynamics, indicating a need for further research. Despite these complex factors, this study demonstrates a significant association between vitamin A and low prevalence of high myopia in men, which is noteworthy.

Additionally, the relationship between oral intake of vitamin A, blood levels, and retinoic acid levels in the choroid is worth considering. Studies have shown that greater oral intake of atRA in chicks actually progressed myopia [28], and non-specific atRA synthesis inhibitors injected into the eye prevented eye elongation [29]. The results of these studies are not consistent with those of our study, and this unexpected discrepancy may be due to the fact that the externally administered atRA was untargeted, or that the non-specific atRA synthesis inhibitor also inhibited other aldehyde dehydrogenases, leading to multi-cellular effects different from those mediated by endogenous atRA. Therefore, it is more important to measure endogenous blood levels without external influences than to gather information on intake. This study analyzed vitamin A based on blood levels, which could be considered a strength of our investigation.

The eye has a blood–retinal barrier, which means that the concentration of certain substances in the serum does not always directly correspond to their concentration inside the eye. Although there is no research directly examining the correlation between serum vitamin A and atRA concentrations in the retina and choroid, one study reported that atRA concentrations in the testes significantly decline when blood vitamin A levels decrease [30]. The blood–testis barrier and blood–retinal barrier are similar in that they both possess tight junctions and selective permeability. Therefore, it can be inferred that a decrease in blood vitamin A levels may lead to a reduction in atRA concentrations within the eye, which could potentially contribute to the development of myopia. However, further research is needed to confirm this hypothesis.

This study has several limitations. Since vitamin A levels were only measured in adults aged ≥20 years, the study findings are applicable only to this age group. Given that ocular elongation primarily ceases during adolescence, the interpretation of our results should be approached with caution. If an adult exhibits low serum vitamin A levels, it is plausible that they may have had similarly low levels during adolescence, assuming no significant lifestyle changes. Based on this assumption, we inferred a potential association between serum vitamin A levels and myopia in this study. However, this remains speculative, and future studies are needed to directly measure serum vitamin A levels in adolescents.

As with all cross-sectional studies, this study assumes that the current exposure reflects the conditions at the time the disease developed, which is an inherent limitation of the study design. Additionally, since some cases of myopia and high myopia continue to progress into adulthood, this study focusing on adults is highly meaningful. In particular, the results related to high myopia are considered a strength.

Additional limitations include the inability to account for genetic, environmental, and socioeconomic factors known to influence high myopia due to the design of this study. Furthermore, measuring refractive power without cycloplegia might introduce errors in myopia prevalence.

## Conclusion

The conclusion of this study is not that serum vitamin A is the cause of myopia, but rather that we identified a relationship where higher serum vitamin A levels were associated with a lower risk of myopia. Further research is necessary to investigate the underlying reasons for this association. Specifically, in men, higher vitamin A levels were related to a low prevalence of high myopia, while, in women, they were associated with a low prevalence of myopia. Increasing blood vitamin A levels could help prevent blindness due to high myopia complications in men and prevent mild myopia in women. Therefore, it is necessary to create an environment that promotes increased blood vitamin A levels.
